# A practical approach to aid physician interpretation of clinically actionable predictive biomarker results in a multi-platform tumor profiling service

**DOI:** 10.3389/fphar.2014.00076

**Published:** 2014-04-16

**Authors:** Kenneth Russell, Leonid Shunyakov, Karel A. Dicke, Todd Maney, Andreas Voss

**Affiliations:** ^1^Caris Life SciencesBasel, Switzerland; ^2^Central Care Cancer CenterBolivar, MO, USA; ^3^Arlington Cancer CenterArlington, TX, USA; ^4^Caris Life SciencesPhoenix, AZ, USA

**Keywords:** evidence-guided, personalized medicine, biomarkers, oncology

## Abstract

Patients in whom the standard of care has failed or who have uncommon tumors for which no standard of care exists are often treated with drugs selected based on the physician’s best guess. The rate of success for this method is generally low. With the advent of fast, affordable tumor profiling technologies, and a growth in the understanding of predictive biomarkers, it is now possible to identify drugs potentially associated with clinical benefit for such patients. We present the Caris approach to evidence-based tumor profiling and two patients with advanced ovarian and prostate cancer in whom standard of care had failed and tumor profiling identified an effective treatment schedule. To establish Caris Molecular Intelligence^TM^ (CMI), over 120,000 clinical publications were screened and graded to characterize the predictive value of biomarkers that form the panel of tests. CMI includes multiple technologies to measure changes in proteins, ribonucleic acid, and deoxyribonucleic acid and proprietary software that matches the test results with the published evidence. The CMI results enable physicians to select drugs that are more likely to benefit the patients, avoid drugs that are not likely to work, and find treatment options that otherwise would not be considered. Worldwide, over 60,000 cancer patients have undergone evidence-based tumor profiling with CMI. In the cases reported in this article, CMI identified treatments that would not have been routinely used in the respective clinical setting. The clinical outcomes observed help to illustrate the utility of this approach.

## INTRODUCTION

Patients who reach the end of their guidelines-defined treatment options and are suitable and willing to receive further treatment, or who present with an uncommon cancer type where treatment options are limited, are among the most difficult to treat. In such cases, the treatment decision is based on the individual patient’s clinical context, physician’s experience and clinical judgment, local practice guidelines and the patient’s medical and treatment history. While the overall rate of success for cancer drug treatment has been estimated at 35% (Jackson, 2009), treatment for these patients is less effective, e.g., the response rate to therapy in patients meeting the inclusion criteria for early clinical studies is around 10% (Olmos et al., 2012).

Advances in the discovery of prognostic and predictive biomarkers can provide oncologists with vital information which helps to stratify their patients for risk of tumor progression and identify potentially beneficial therapeutic agents based on biomarker expression patterns. For example, lung cancer has traditionally been viewed as difficult to treat and associated with poor prognosis. The last 5 years has seen epidermal growth factor receptor (EGFR) mutation testing become standard of care for selection of treatment with erlotinib or gefitinib in non-small cell lung cancer (NSCLC). Many companion diagnostics are now part of the approved drug label and clinical guidelines indicate which specific biomarker should be assessed in a consistent manner in all patients prior to treatment selection in certain tumor types in order to identify a defined subgroup for which the respective treatment is indicated.

While many new drugs received regulatory approval together with companion diagnostics in a limited, often lineage specific, clinical setting, the biological principles governing cancer growth can often be extrapolated to other indications as well. One example is the utility of human EGFR 2 (HER2)-directed treatments for patients with lung cancer that have a HER2 (encoded by the *ERBB2* gene) mutation. While this occurs in fewer than 2% of all patients with lung cancer HER2-directed targeted treatments led to disease control in 82% of patients with HER2 mutations (Mazières et al., 2013). This shows that for a patient with no available standard treatment options molecular profiling can reveal specific biomarkers that are associated with benefit from drugs that would typically not have been considered for treatment.

As the majority of individual mutations or other molecular changes are usually rare, a comprehensive profiling increases the chance that a valuable alteration is identified. Comprehensive profiling delivers all relevant information at once, rather than taking a stepwise approach where tests are ordered one by one dependent on the outcome of single biomarker results. This saves valuable time for the patient and confers an important advantage because the comprehensive overview of the patient’s molecular changes provides the best support for rational treatment decisions.

Over recent years, tumor profiling has become a standard in many large university centers. Tsimberidou et al. (2012) published how tumor profiling guided recruitment of patients into clinical trials with targeted drugs at the MD Anderson Cancer Center. Patients enrolled in studies that required a matching genetic aberration had a clinical response rate of 27% whereas only 5% of patients that could not be assigned to a trial based on molecular profiling responded (Tsimberidou et al., 2012). In a similar approach undertaken at the Princess Margaret Cancer Centre in Toronto, six of twenty-one patients (29%) enrolled into ongoing clinical trials with a therapy matched to a genetic aberration had a confirmed partial response (Bedard et al., 2013). Both of these groups employed large, cross-functional teams of experts to interpret the results from molecular testing. The ability to dedicate experienced teams to guide tumor profiling-directed treatment is beyond the capabilities of most community practices. Therefore alternative methods had to be developed to allow patient’s access to reliable and actionable tumor profiling results.

## CMI: AN EVIDENCE-BASED APPROACH OF TUMOR PROFILING FOR CLINICAL DECISION-MAKING

Caris Life Sciences started to offer evidence-based molecular profiling over 8 years ago. The approach taken has been constantly refined, resulting in a service which is adapted to the latest scientific knowledge. Caris Molecular Intelligence^TM^ (CMI) supports physicians in implementing actionable results from comprehensive tumor profiling in their routine practice. CMI is performed in a high-throughput laboratory that has been customized to accommodate large numbers of specimens for testing on multiple technology platforms. Comprehensive molecular testing is coupled with an evidence-based proprietary algorithm that translates complex biomarker results into a table of drugs that may provide benefit or lack of benefit for that patient. Caris has received accreditation from Clinical Laboratory Improvement Amendments (CLIAs), as well as an extensive list of certifications from the state of New York. Caris also has recently obtained the molecular profiling industry’s first-ever accreditation to the International Standards Organization (ISO) 15189: 2012 “Medical laboratories – Requirements for quality and competence,” by the American Association for Laboratory Accreditation (A2LA). The CMI service provides each treating physician with the relevant biomarker testing and current expert interpretation needed to make clinical treatment decisions for each patient.

The CMI service uses a variety of established technology platforms to measure a panel of carefully selected biomarkers including immunohistochemistry (IHC), fluorescent *in situ* hybridization (FISH), polymerase chain reaction (PCR), and direct gene sequencing. Taking an approach that is not reliant on a single technology is critical to perform clinically relevant biomarker testing. Proteins, gene expression, mutations, and gene rearrangements can all have utility as predictive biomarkers. For example, of the 93.5% of compounds predicted to be beneficial which were identified, 87.2% of them were driven by IHC and ISH results, 12.6% by IHC, ISH, and NGS results, and 0.2% driven by NGS results alone. Therefore, an assessment of the molecular profile of a tumor with just one technology will miss potential therapeutic options for the patient.

**FIGURE 1:**
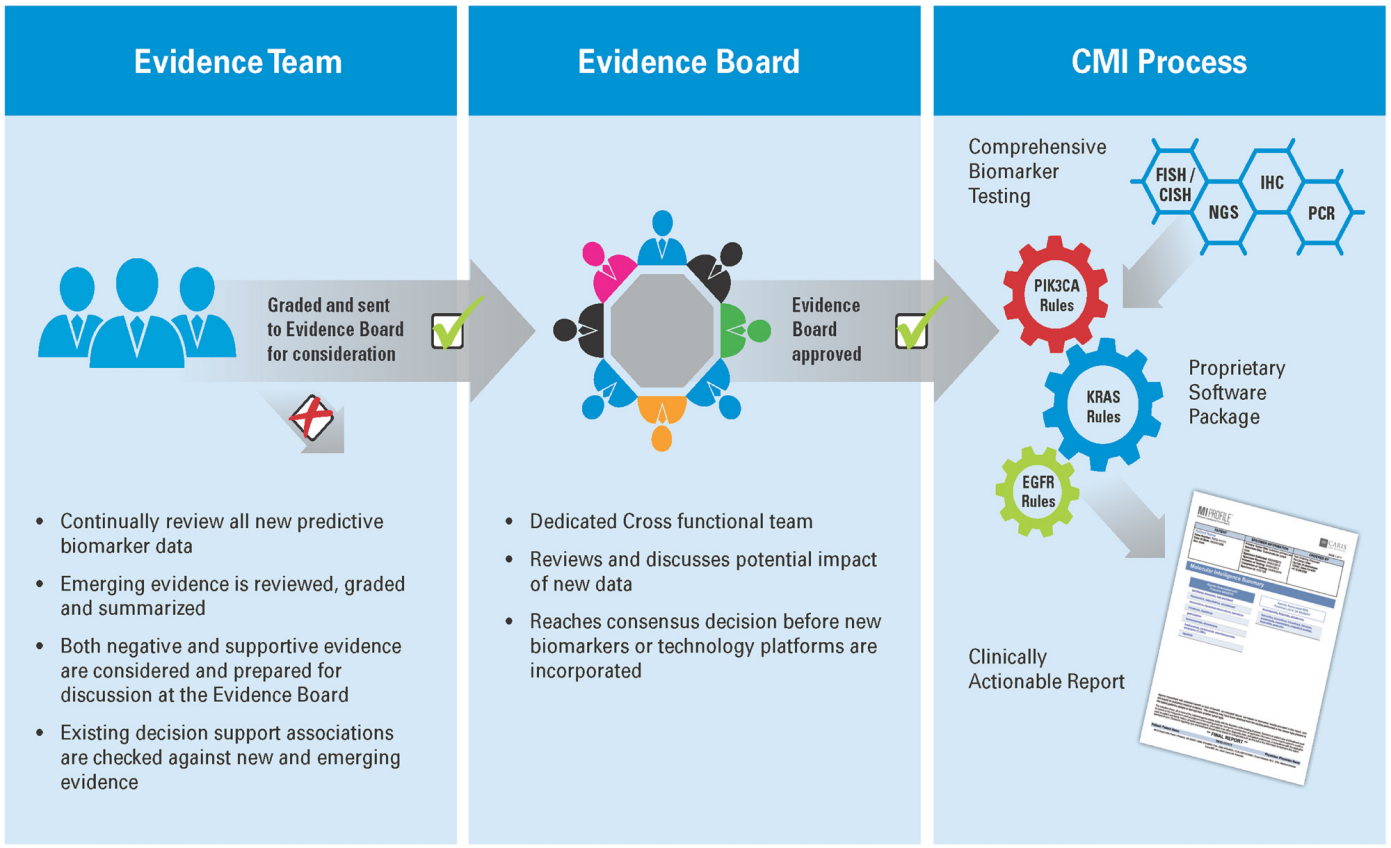
**Generating Molecular Intelligence: an extensive literature review is performed by a multi-disciplinary review team, which examines new research and relevant research, and grades it based on US Preventative Task Force methodology in the context of the biomarkers revealed through the profiling services.** The output from this evidence review forms the basis of a proprietary software package which is used to translate the results of comprehensive biomarker testing to a clinically actionable report, providing physicians with meaningful biomarkers, actionable drug associations, and relevant clinical trials for their individual patient.

To keep the biomarker panel current, an ongoing review of the medical literature is performed to review the evidence of predictive associations of biomarkers with available therapeutics. The interpretation of the biomarker evidence is under the governance of a cross-functional group comprised of oncologists, molecular geneticists, pathologists, and research scientists. All biomarkers tested in the CMI service are included based on the strength of their supporting evidence according to a version of the United States Preventative Services Task Force (USPSTF) level of evidence methodology adapted from Harris et al. (2001). The content of each scientific paper is appraised for study design, study validity, and applicability of the biomarker in drug selection. Today, over 95% of drug/biomarker associations included in the service are supported by level 1 (randomized, controlled trials or meta-analyzes) or level 2 (non-randomized, controlled trials, single arm or cohort/case-control analytic studies) evidence. The evidence system used by Caris allows the service to evolve in response to new clinical data. As new markers are identified and vetted, they can be added to the panel quickly. An overview of the evidence process for CMI is shown in **Figure 1**.

The CMI report aligns the molecular profile of the patient’s tumor to relevant therapeutic agents associated with potential benefit or potential lack of benefit for the purpose of serving as a decision support aid for the treating physician. The report results are provided to the treating physician and supported by the relevant references from the peer-reviewed literature as determined by the evidence review process. The report also enables the treating clinician to review in detail the biomarker testing that has been performed, as well as link directly to the clinical evidence supporting the biomarker-drug association.

## CLINICAL EVIDENCE SUPPORTING USE OF CMI

A manuscript has been published with a number of independent accompanying abstracts which have reported how CMI was used in clinical practice. The Bisgrove study (Von Hoff et al., 2010) was the first clinical trial to assess the use of a multi-platform approach to molecular profiling to identify treatment targets in patients with refractory cancers. In this study, patients were profiled with the CMI panel and physicians chose a treatment regimen based on the results. Clinical benefit was defined as a 30% increase in progression-free-survival (PFS) with molecularly guided treatments, compared to the PFS under the most recent prior regimen. The majority of patients had molecularly identifiable targets and 18 of 66 patients (27%) treated on the basis of molecular profiling derived clinical benefit. A recent study in patients with refractory breast cancer showed that tumor profiling resulted in a revision of the original treatment decision for all patients. Tumor profiling based therapy resulted in a clinical benefit in 52% of heavily pretreated patients (Jameson et al., 2013). A review of all patients treated in a single center in Australia resulted in clinical and survival benefits in over half of the patients and confirmed the role of molecular profiling in a clinical practice setting (Dean and Wallace, 2013). Though preliminary evidence supports clinical utility, the degree to which CMI improves patient outcomes has not yet been demonstrated conclusively. Further evaluations of the approach are currently ongoing.

## CASE STUDY: RESPONSE TO PEMETREXED IN A PATIENT WITH METASTATIC PROSTATE CANCER

A 63-year-old patient was diagnosed with metastatic prostate cancer in 2006 with a Gleason score of 9 and involvement of the pelvic lymph nodes. The patient initially received radiation therapy to the pelvis and the prostate followed by treatment with gonadotropin-releasing hormone (GnRH) analogs. After 4 years the disease progressed and metastasized to the bones, lung, and liver. Treatment with bisphosphonates, sipuleucel-T, abiraterone, enzalutamide, docetaxel, and cabazitaxel did not stop progression. The disease could never be controlled except for short transient partial responses under docetaxel and abiraterone. Embolization of the left liver lobe resulted in temporary local pain relief and as a last resort, the patient received carboplatin with etoposide, which resulted in a transient partial response followed by rapidly progressive disease.

As all guideline-recommended treatment options had failed, it was decided to perform CMI tumor profiling. At this time, the patient had extensive liver metastases with underlying cirrhosis, which caused considerable pain. He was wheelchair bound and unable to walk with an Eastern Cooperative Oncology Group (ECOG) performance status of 3, approaching 4. Within 6 weeks, his prostate specific antigen (PSA) had risen from 84 ng/ml to 177 ng/ml and lactate dehydrogenase (LDH) levels had risen from 569 to 2196 IU/L. All signs and symptoms pointed to a rapidly progressive decline of the patient’s general condition.

**FIGURE 2:**
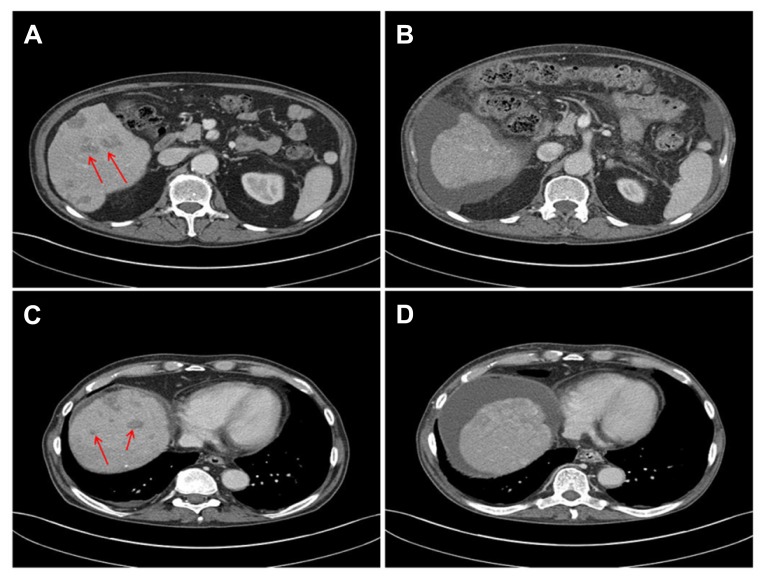
**CT scans before (A,C) and 5 weeks after initiation of treatment with pemetrexed (B,D) show decrease in size of metastases and liver sclerosis and ascites due to previous embolization**.

The CMI report indicated that the tumor was not expressing thymidylate synthase (TS), a protein involved in generation of critical components for DNA synthesis and repair pathways. Published level II evidence from a study of 268 patients with advanced NSCLC who received treatment with pemetrexed after prior chemotherapy found that patients with low TS expression had a longer median PFS compared to those with high TS expression (Chen et al., 2011). The CMI report associated low TS expression with tumor sensitivity to fluoropyrimidines and other folate analogs with potential benefit from 5-fluorouracil, capecitabine or pemetrexed. Therefore, it was decided to begin treatment with single agent pemetrexed at the end of September 2013 based on the physician’s choice. The patient’s general condition improved rapidly and the intense right upper quadrant pain resolved completely. Within 5 weeks of starting pemetrexed treatment, a computed tomography (CT) scan showed decrease in size of multiple liver metastases (**Figure 2**). The tumor marker PSA dropped to 5.1 ng/mL and LDH returned to normal (423 IU/L). The patient can now care for himself and his ECOG performance status is 1. The treatment has been tolerated exceptionally well and no further admissions to the hospital became necessary. After 4 months, at this time of this report, the patient continues to receive 3 weekly cycles of pemetrexed, PSA (7.5 ng/mL) and LDH (539 IU/L) are indicating a continued response.

## CASE STUDY: RESPONSE TO CETUXIMAB AND IRINOTECAN IN A PATIENT WITH METASTATIC OVARIAN CANCER

A 49-year-old woman was diagnosed with stage IV ovarian cancer in August 2009 after feeling abdominal pain. A CT scan revealed that she had a mass in her right ovary, which was diagnosed as a mixed high grade serous and endometrioid carcinoma. Surgery confirmed metastatic disease and the patient began standard treatment with a combination of intraveneous paclitaxel and carboplatin and intraperitoneal docetaxel/cisplatin. During the time on treatment, the patient had a partial response; her cancer antigen 125 (CA-125) level dropped from 475 to 70 U/mL but did not return to normal levels (less than 35 U/mL). A laparotomy revealed no obvious tumor masses but the persisting elevation of CA-125 indicated residual disease.

As the standard treatments had failed a portion of the initial biopsy material was sent for CMI testing to identify additional treatment options. Based on the findings of the report, the patient was treated with doxorubicin followed by topotecan but both treatments had to be discontinued due to intolerable toxicities. Doxorubicin was selected based on overexpression of topoisomerase 2A (TOP2A), which has been linked with level II evidence to doxorubicin response in breast cancer (Durbecq et al., 2004). In patients with ovarian cancer treated with topotecan, tumors with low or undetectable Topoisomerase 1 (TOPO1) protein levels had a median time to progression of 4.3 months, compared to 13.2 months in patients with high TOPO1 expressing tumors (Litzow et al., 2010). Although poorly tolerated, both treatments resulted in transient decreases in CA-125 to normal levels.

The CMI report also found overexpression of the *EGFR* gene and overexpression of the TOPO1 protein. EGFR gene overexpression along with high phosphatase and tensin homolog (PTEN) protein expression indicated potential efficacy from cetuximab, which is targeting the EGFR receptor (Personeni et al., 2008; Sartore-Bianchi et al., 2009). Irinotecan causes cell killing by blocking TOPO1. In addition to the topotecan evidence cited above, level II evidence in colorectal cancer (CRC) patients treated with irinotecan-containing adjuvant chemotherapy demonstrated that there was a significant improvement in survival in patients who expressed TOPO1 compared to those with low TOPO1 expression (Kostopoulos et al., 2009). These results indicated potential benefit from cetuximab and irinotecan and combination treatment with these drugs was started in late October 2010. After 2 months, bevacizumab was transiently added to this combination (based on overexpression of the hypoxia-inducible factor 1-alpha (*HIF1A*) gene) until it caused toxicity. Within 2 months of the starting the combination of cetuximab and irinotecan the patient’s CA-125 level dropped from 64 to 10 and stayed normal over the course of the first 8 months of treatment. Although an attenuated dose was used, toxicities led to discontinuation of therapy. After discontinuation of cetuximab and irinotecan the patient developed progressive disease with pelvic and liver metastases. The patient was then put on treatment with cyclophosphamide, which was not included on the CMI report but resulted in a transient response. Currently the disease is slowly progressive and the patient is still on cyclophosphamide at an attenuated dose.

A significantly long remission after recurrence is unusual in ovarian cancer and irinotecan and cetuximab are rarely used in ovarian cancer. This observation is of significant importance as it justifies further exploration of treatments guided by tumor profiling instead of using histological diagnosis of the tumor alone.

## CONCLUSION

Patients with metastatic cancer frequently arrive to a point in their clinical care when all standard of care options have been tried and they require further treatment. Although guidelines recommend that these patients enter either clinical trials or palliative care, they are often fit enough and willing to continue to receive further cytotoxic treatment. Comprehensive tumor profiling can identify active treatment options, help avoiding treatments which are likely not active, and find treatments that otherwise would not have been considered. Meaningful integration of the information generated by comprehensive biomarker testing requires cross-functional expertise to aid interpretation and determine which results are clinically relevant. As this level of support is rarely accessible, CMI provides a service which helps physicians to develop evidence-based treatment plans. CMI combines the results of tumor profiling with a thorough assessment of the published clinical evidence in a comprehensive report that includes drugs associated with benefit, lack of benefit as well clinical trials that may be relevant for the patient. The clinical experience with tumor profiling in routine clinical practice has been promising.

## Conflict of Interest Statement

Kenneth Russell, Andreas Voss, and Todd Maney are employees of Caris Life Sciences. Drs. Shunyakov and Dicke did not have any commercial or financial relationships that could be construed as potential conflicts of interest.
